# SDM: a server for predicting effects of mutations on protein stability

**DOI:** 10.1093/nar/gkx439

**Published:** 2017-05-19

**Authors:** Arun Prasad Pandurangan, Bernardo Ochoa-Montaño, David B. Ascher, Tom L. Blundell

**Affiliations:** 1 Department of Biochemistry, University of Cambridge, Cambridge CB2 1GA, UK; 2 Department of Biochemistry and Molecular Biology, University of Melbourne, Australia

## Abstract

Here, we report a webserver for the improved SDM, used for predicting the effects of mutations on protein stability. As a pioneering knowledge-based approach, SDM has been highlighted as the most appropriate method to use in combination with many other approaches. We have updated the environment-specific amino-acid substitution tables based on the current expanded PDB (a 5-fold increase in information), and introduced new residue-conformation and interaction parameters, including packing density and residue depth. The updated server has been extensively tested using a benchmark containing 2690 point mutations from 132 different protein structures. The revised method correlates well against the hypothetical reverse mutations, better than comparable methods built using machine-learning approaches, highlighting the strength of our knowledge-based approach for identifying stabilising mutations. Given a PDB file (a Protein Data Bank file format containing the 3D coordinates of the protein atoms), and a point mutation, the server calculates the stability difference score between the wildtype and mutant protein. The server is available at http://structure.bioc.cam.ac.uk/sdm2

## INTRODUCTION

Recent developments in next-generation sequencing methods have provided a wealth of information on the genetic mutations present in different organisms. In humans, >100 000 genetic variants have been statistically associated with disease conditions (1). The big challenge is to identify and characterize those genetic mutations that have functional consequences. Of particular interest are missense mutations that can disrupt functions of proteins by modulating their stability as well as affecting interactions with other biological molecules. Hence, predicting the impacts of mutations on protein stability and interactions is fundamental to the understanding of various biological processes, including disease and drug resistance (2).

While experimental techniques to measure changes in stability between wild-type and mutant proteins are the most accurate, they are time consuming and costly (3). There is a strong need for the development of computational techniques to predict the impacts of mutations on protein stability in order to support the rapid and routine analysis of sequencing data necessary for personalized medicine (4).

Methods to predict the impacts of mutations can be broadly classified into sequence- and structure-based approaches. Various sequence-based methods using a broad range of methods have been reported, including support vector machine (INPS) (5), neural networks (6) and decision trees (iPTREE-STAB and MuStab) (7,8). Structure-based methods use either machine learning techniques (9–16) or potential-energy-based approaches to predict the impacts of mutations (17–19). Multi-agent prediction systems, based on statistical scoring functions and machine-learning approaches, have also been reported (20). Various other predictive methods have recently been reviewed elsewhere (21). The development and validation of various computation methods is also supported by databases documenting experimental thermodynamic parameters, including the change in free energy between the wild-type and mutant protein (22,23).

Here, we report our updated knowledge-based approach SDM (24,25) and its webserver SDM2 for predicting the effects of mutations on protein stability. SDM pioneered the use of conformationally constrained environment-specific substitution tables (ESSTs) to calculate the change in thermal stability between the wildtype and mutant protein (24–27). SDM has also been successfully used in combination with machine learning techniques to predict better the impact of mutations on protein stability (15). Unlike machine learning methods, SDM predictions do not rely on a number of features for training and do not suffer from the issue of overfitting. SDM2 uses newly recalculated environment-specific substitution tables (ESSTs) for the purpose of calculating the stability difference score between the wildtype and mutant protein structures. New structural parameters, based on residue packing density, have been introduced into the calculation of ESSTs. The newly updated ESSTs were derived from a large set of protein-family sequence and structure alignments, reflecting the current state of fold-classification databases. Below we describe the updated method, webserver and results of the validation process for three different benchmark datasets.

## MATERIALS AND METHODS

### Environmental-specific substitution tables

SDM uses a set of conformationally-constrained ESSTs to calculate the difference in stability between the wildtype and mutant protein structure (26,27). In SDM2, the updated ESSTs were derived from 2054 protein family sequence and structure alignments from the TOCCATA database, originally developed to serve as a resource for template identification in homology modelling (28) (Ochoa-Montaño B, and Blundell TL, manuscript in preparation), consisting of 12 038 structures. The TOCCATA database incorporates all domains from SCOP 1.75A and CATH 3.5, forming a consensus ‘profile’ whenever the domains of a SCOP family can be reasonably matched to a CATH superfamily, otherwise keeping them in their respective categories.

For the calculation of ESSTs we took representative crystal structures (better than 2.5 Å resolution) within each family by following the sequence-clustering procedure using Cd-hit (29), as previously described (30). The program ULLA was used for the purpose of calculating ESSTs (31). ESSTs take the form of probability tables giving details about the amino acid residue conservation and substitution to any other residue occurring in a well-defined local structural environment. They have been shown to capture distinct substitution patterns, specific to a given local structural environment (32). Functional residues, defined as those involved in catalytic site, ligand binding and protein-protein interactions, were identified using CREDO (33) and masked from substitution counts.

Previously, in SDM the ESSTs were derived from HOMSTRAD (34) using 371 protein family sequence alignments consisting of 1357 structures. In SDM2, the ESSTs derived from TOCCATA represent a 6- and 9-fold increase in the number of protein families and structures respectively.

In this paper, we used two further structural parameters based on residue-occluded packing density (OSP) (35,36) and residue depth (37,38) as alternatives to the relative sidechain solvent accessibility (RSA) (39) parameter used in the calculation of ESSTs. The occluded surface for a given residue represents the molecular surface of the surrounding non-bonded atoms found within 2.8 Å (33,34). The OSP of a residue is calculated as a function of occluded surface area and average unit normal distances between the molecular surfaces of the atoms in a given residue and the neighbouring van der Waals surfaces. The depth of a residue is defined as the average distance of all atom depths found in the residue from the nearest surface water molecule (37,38). RSA can help identify whether a residue is solvent accessible or inaccessible. We have previously proposed that OSP and residue depth could be used to classify better the environment of the interior of the protein (40).

OSP and residue depth have been shown to be important in protein structure and stability analysis (37,41,42). Previous studies have shown that the free energy difference between wild-type and mutant proteins (ΔΔ*G*) correlates better with changes in packing parameters such as occluded surface or residue depth than with the change in accessible surface area upon folding (43). Our analyses of the distribution of OSP, depth and RSA based on the TOCCATA dataset suggest that both OSP and depth could be useful structural parameters in defining ESSTs (see Supplementary material, Supplementary Figures S1 and S2). Our recent analysis of ESSTs using the TOCCATA database indicate that the residue conservation progressively increases with residue depth and packing density and could serve as a good indicator for the classification of disease and non-disease mutations (40). These results suggest that accounting for packing interactions is crucial for understanding the energetics of protein mutant stability.

We have used various structural parameters to define the local structural environment of amino acid residues for the purpose of calculating ESSTs (See Supplementary text for the description of the individual local structural environments). In SDM2, we used a set of 216 ESSTs defined by the combination of the local structural environment parameters (nine main-chain conformations  ×  three residue occluded surface packings  ×  eight hydrogen bonding) (see Supplementary text). The previous version of the SDM webserver (25) used nine main-chain conformations, three RSA classes and only two classes of hydrogen bonding, based on the satisfaction of hydrogen bonding potential (44), resulting in a total of 54 ESSTs (denoted 54_RSA, see Supplementary text).

For the purpose of testing SDM2, we have also calculated different sets of substitution tables using residue depth (216_depth), a combination of OSP and residue depth (648_RSA_OSP_depth) as well as a set of ESSTs based on RSA (54_RSA) (see Supplementary text).

### Prediction of the impact of mutations on protein stability

The stability difference score in SDM is calculated as follows.

(1) \begin{equation*}\Delta \Delta s = \Delta s_{jk}^U - \Delta s_{jk}^F - \Delta s_{jk}^{Disrupt}\end{equation*}

where $\Delta s_{jk}^U$ and $\Delta s_{jk}^F$ are the differences in stability scores associated with the substitution of residue type *j* by *k* in the unfolded and folded states respectively. They are calculated using ESSTs as described elsewhere (24,25).

In addition to the disruption term $\Delta s_{jk}^{Disrupt}$ described in the original method (24), in SDM2 we have included a new penalty function for the substitution of buried bulky hydrophobic residues (Phe, Leu and Ile) by Ala or Val that have relatively non-bulky sidechains. All residues with RSA <17% are considered to be buried. The cutoff of 17% was chosen on the basis of an assessment of relative sidechain solvent accessibility values (45). Residue substitutions creating void volumes in the buried region of the protein are better quantified using changes in OSP than changes in sidechain surface accessible area. The newly designed cavity penalty function uses a similar form of disruption term. Instead of accounting for the absolute value of the net change at the mutated position in the sidechain surface accessible area, we modelled it using the absolute value of the net change between the OSP of wild-type and mutated residue relative to the average OSP values (0.33) (36) found at solvent exposed regions of the protein. The weighting used in the logarithmic function is adjusted accordingly to improve the stability prediction for buried cavity forming mutants. Our analysis and stability prediction using the large mutant dataset showed considerable improvement in the quality of predictions when using the newly introduced cavity penalty term in addition to the disruption term (see Validation section). Therefore, the SDM2 stability difference score in Equation (1) becomes

(2) \begin{equation*}\Delta \Delta s = \Delta s_{jk}^U - \Delta s_{jk}^F - (\Delta s_{jk}^{Disrupt} + \Delta s_{jk}^{Cavity})\end{equation*}

### Hypothetical reverse mutations

Since the folding free energy (ΔΔ*G*) is a thermodynamic state function, the ΔΔ*G* of a mutation from a wild type protein to its mutant (ΔΔ*G*_wt→mut_) equals the –ΔΔG of a hypothetical reversed mutation from the mutant to the wild type protein, ΔΔ*G*_mut→wt_. In this study, we also considered the hypothetical reverse mutations in order to test the robustness of the SDM method for predicting protein stability changes upon mutations.

### Mutant thermodynamic dataset

For the purpose of testing the method, we have used the following datasets containing only single point mutations. These datasets contain experimental thermodynamic parameters for wildtype and mutant proteins, including the change in Gibbs free energy (ΔΔ*G*).

#### S2648

The first data set, S2648 (17), derived from the ProTherm database (22), comprises 2648 single-point mutations in 131 different globular proteins.

#### S350

The second data set, S350 (17), is a randomly selected subset of the S2648 dataset comprising 350 mutations in 67 different proteins. We also use this dataset to compare the performance of SDM with other methods.

#### p53

This dataset contains 42 mutations within the DNA binding domain of the tumour suppressor protein p53 (12). This protein has been extensively studied and the experimental ΔΔ*G* values were obtained from the literature (46–50).

#### S140

In order to test the prediction of hypothetical mutations we considered using the dataset taken from Li *et al*. (51). It contains 140 single point mutations with known 3D structures for both wildtype and mutant proteins and comprises a total of 128 mutations unique to this dataset.

## WEBSERVER

### Input

The server provides two different input options for the user. The ‘Single Mutation’ option allows the user to predict the effect of a single mutation on the stability of the protein. This option accepts a PDB file or a PDB code and the point mutation specified as a string containing the single letter code of the wildtype residue in the protein, its corresponding residue number and the single-letter code of the mutant residue. The newly introduced ‘Mutation list’ option allows the user to upload an input file containing a list of up to 20 mutations (input string format similar to the first option), with each mutation listed on a separate line. For both input options the user has to specify the chain id of the protein relevant to the mutation. In addition, the server allows the user to predict the stability score for a reverse mutation by selection of the ‘Predict reverse mutation’ checkbox. Pressing the ‘Run SDM’ button will start the processing on the server.

### Output

**Figure 1.**
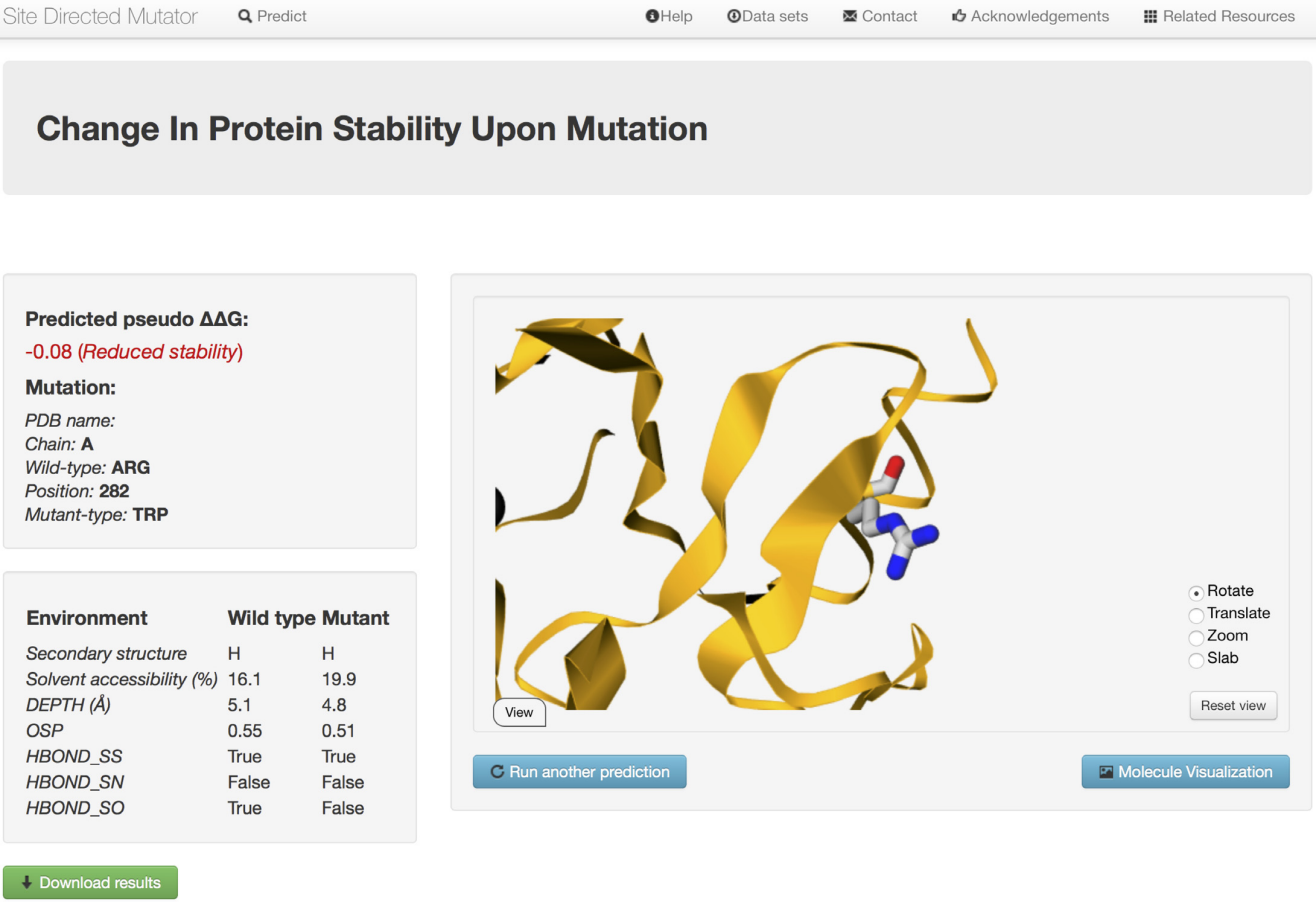
Snapshot of the SDM2 output page, showing the prediction results for the R282W mutation in the tumour suppressor protein p53 (PDB code 2OCJ, chain A). The two left panels display the details of the input mutation, SDM2 stability prediction score for the mutation as stabilising or destabilising and the properties of the structural environment (mainchain conformation class, sidechain solvent accessibility, side chain hydrogen bonding pattern, residue depth and packing density) along with its values for wildtype and mutant residues. The output page also allows the visualisation of the input protein with the wildtype residue (shown in stick representation) and its surrounding protein environment. The user can also download the results as a text file along with the model of the mutant proteins. In p53, the R282W mutation is shown to destabilise the protein resulting in p53 being largely unfolded and inactive (47). SDM2 predicts this mutation to be destabilising and also show considerable improvement over the previous version SDM which predicts this mutation as highly stabilising (25).

For the ‘Single Mutation’ option, the webserver outputs the predicted stability difference score (pseudo ΔΔ*G*). The negative (in red) and positive (in blue) values correspond to mutations predicted to be destabilising and stabilising, respectively. A summary of the input is presented, highlighting the wild-type residue, residue number, chain and the mutant residue. A separate panel lists various structural features used in SDM2 prediction including the class of mainchain conformation, sidechain solvent accessibility, sidechain hydrogen bonding pattern for the wildtype and mutant residues. In the updated server, we have added two new structural annotations including the residue depth and packing density to improve the prediction of stability. The uploaded PDB file with its wildtype residue environment can be visualised directly from the server using the GLmol molecular viewer (Figure 1). For the input ‘Mutation list’, the server output (content similar to the ‘Single Mutations’ option) is shown in table format. The user can download prediction results as comma-separated text files and models of the mutated protein for further analysis.

## VALIDATION

For 95 stabilizing (ΔΔ*G* ≥ 0.0) and 255 destabilising (ΔΔG < 0.0) mutations of the widely used S350 dataset, as well as 42 mutations in the p53 tumor suppressor (11 stabilizing and 31 destabilising), SDM2 achieved Pearson correlations of 0.61 and 0.68 to the experimental observations, a significant improvement of 24% and 134% respectively from the previous version (25) (Table 1). For both S350 and p53 the accuracy and Matthews correlation coefficient were found to be above 0.71 and 0.31 respectively (Table 1). For the largest benchmark S2648 (602 stabilising and 2046 destabilising mutations), the Pearson correlations, accuracy and Matthews correlation coefficient were 0.48, 0.71 and 0.29 respectively. This represents an improvement in correlation of 7% over the previous version of SDM (Table 1). For all cases, the standard error was within 1.56, with S350 achieving the lowest standard error of 1.29 (Table 1).

**Table 1.** Performance of SDM2 on the datasets using new ESSTs based on residue packing density

Dataset	*R* ^a^ (SDM2/SDM)^d^	Accuracy (SDM2/SDM)^d^	MCC (SDM2/SDM)^d^	σ (SDM2/SDM)^d^
P53	0.68/0.29	0.76/0.62	0.31/0.07	1.56/2.12
S350	0.61/0.49	0.71/0.66	0.33/0.30	1.29/1.86
S309^b^	0.61/0.50	0.73/0.68	0.34/0.30	1.32/1.86
S87^c^	0.69/0.61	0.93/0.87	0.73/0.61	1.71/2.16
S2648	0.48/0.45	0.71/0.67	0.29/0.28	1.46/1.79

^a^Pearson product-moment correlation coefficient.

^b^S309 is a subset of S350 containing 309 mutants with ΔΔ*G* prediction available for all predictors.

^c^S87 is a subset of S350 containing 87 mutants with the experimental ΔΔ*G* values causes >2 kcal/mol change and for which a ΔΔ*G* prediction is available from all predictors.

^d^Values are shown for the updated SDM2 in comparison with the previous version of SDM separated by slash.

**σ** is the standard error; MCC is the Matthews Correlation Coefficient.

Please see Supplementary text for details about the calculation of Pearson product-moment correlation coefficient, Accuracy, MCC and **σ**.

We also tested the method with various sets of ESSTs that included different combinations of structural parameters (Supplementary Table S1). The results showed that the ESSTs 216_RSA, 216_depth and 648_RSA_OSP_depth performed equally well in comparison with the 216_OSP that is currently used as default by the method (Supplementary Table S1). In most of the cases, the accuracy and Matthews correlation coefficients calculated for S350, P53 and S2648 using ESST 216_OSP are marginally better than 216_RSA, 216_depth and 648_RSA_OSP_depth (Table 1, Supplementary Table S1). The ESST set previously used by the SDM webserver, 54_RSA (25), was the poorest performing among the tested ESSTs. It is worth mentioning that for the commonly found destabilizing mutation in p53 (R282W), SDM2 was able to predict and classify it as a destabilising mutation (pseudo ΔΔ*G* = –0.08), whereas the previous version of SDM wrongly predicted it as a highly stabilising mutation (pseudo ΔΔ*G* = 3.50).

To demonstrate the impact of the cavity penalty, we performed the SDM2 prediction for all the datasets (shown in Table 1) without the cavity penalty contribution. The results show that the removal of cavity penalty considerably reduced the Pearson correlation of all the datasets (Supplementary Table S2), suggesting the importance of its inclusion.

We used the dataset S350 for the purpose of comparing SDM2 with other methods. We compared the performance of SDM2 with eight different methods that employ various techniques, including knowledge-based, energy-based and machine-learning approaches. The results show SDM is one of the top performing methods (Table 2).

**Table 2.** Comparison of the performance of different prediction methods

		S350/S309/S87^b^	
Method	No. of predictions^a^	*R* ^c^	σ (kcal/mol)
SDM2	350	0.61/0.61/0.69	1.29/1.32/1.71
AUTOMUTE	315	0.46/0.45/0.45	1.43/1.46/1.99
CUPSAT	346	0.37/0.35/0.50	1.91/1.96/2.14
Dmutant	350	0.48/0.47/0.57	1.81/1.87/2.31
Eris	334	0.35/0.34/0.49	4.21/4.28/3.91
I-Mutant-2.0	346	0.29/0.27/0.27	1.65/1.69/2.39
PopMuSic-2.0	350	0.67/0.67/0.71	1.61/1.19/1.67
mCSM	350	0.73/0.74/0.82	1.08/1.10/1.48
MAESTRO	350	0.70/0.69/0.76	1.13/1.17/1.67

^a^350 mutations were tested with each method. However, some servers failed to compute the ΔΔ*G* prediction for all mutants, resulting in predictions for less than the full number.

^b^Three values are shown separated by slash. The first value corresponds to the whole validation set of 350 mutants. The second value corresponds to the 309 mutants with ΔΔ*G* prediction available for all predictors. The third value corresponds to 87 mutants with the experimental ΔΔ*G* values causes >2 kcal/mol change and for which a ΔΔ*G* prediction is available from all predictors.

^c^Pearson product-moment correlation coefficient.

### Analysis of residue depth and packing density in mutant stability dataset

We used the largest mutant dataset, S2648, to analyse the distribution of residue depth and packing density found in highly stabilising (ΔΔ*G* > 2.5 kcal/mol) and destabilising mutations (ΔΔ*G* > –2.5 kcal/mol). The analysis shows that the highly destabilising mutations are mostly found at high residue packing density regions (OSP > 0.56) and occur at two distinct depth levels (4 and 8 Å) (Supplementary Figure S3A and C). Previously, it has been predicted that the damaging mutations have a higher probability of occurring at the protein interior (52). However, highly stabilising mutations were observed to occur mostly at high packing density regions and at residue depth ∼4 Å (Supplementary Figure S3B and D).

We also used the dataset S2648 to study the impact of the cavity creating hydrophobic mutations on protein stability as well as the structural signatures (accessibility, depth and packing density) associated with such mutations. About 9% of the mutations in S2648 (226/2648) are found to be cavity-forming hydrophobic mutations. The minimum, maximum and the average of the experimental ΔΔ*G* values for those mutations are –5.0, 2.1 and –2.1 kcal/mol respectively, showing that most of the cavity forming mutations are highly destabilising in nature with a standard deviation of 1.40. For those mutations, the average cavity penalty contribution is 1.65 which is in scale with the average experimental ΔΔ*G* values. From the structural environment point of view, the average values of relative sidechain solvent accessibility, packing density and depth of the wildtype residues involved in cavity forming mutation are 3%, 0.50 and 7.12 Å respectively. For cavity forming hydrophobic mutations, the residue depth shows the most variation (σ = 1.68) compared to the residue-packing density (σ = 0.07).

### Prediction using hypothetical reverse mutations

As discussed earlier, the ΔΔ*G* of a mutation from a wildtype protein to its mutant (ΔΔ*G*_wt→mut_) is equivalent to the –ΔΔ*G* of a hypothetical reverse mutation from the mutant to the wild type protein, ΔΔ*G*_mut→wt_ (51). To test this, we used the dataset S140 to conduct an evaluation of the performance for reverse mutations (assuming no conformational changes involved in the generation of the mutant models). The dataset contains experimental ΔΔ*G*'s for 140 mutations with known 3D structures for both wildtype and mutants. SDM2 prediction shows positive correlation for both forward (0.50) and reverse mutations (0.19) (Table 3, Supplementary Table S3). The performance of SDM2 to predict forward and reverse mutations was compared with the previously reported comparison study using three different methods (51). The method PROTS (51) shows comparatively strong correlation for both forward and reverse mutations (Table 3). However, the prediction performance of the machine-learning approaches like MUpro (11) and I-Mutant2.0 (10), diminishes for the hypothetical reversed mutations (Table 3). The sequence based method INPS (5) that uses a Support Vector Machine algorithm has been shown to predict reverse mutations better than SDM2 for the largest dataset S2648. The Pearson's correlation coefficients of the former and latter are 0.53 and 0.24 respectively. We observed that for dataset S140, the removal of the disruption penalty from SDM2 further improved the overall prediction of the hypothetical reverse mutation with the correlation for forward and reverse mutations equal to 0.42 and 0.39 respectively (Supplementary Table S3). Similar trends were observed when tested on several other benchmark sets including p53, S350 and S2648 that require the generation of mutant models (Supplementary Table S3). It is also worth noting that the removal of the disruption penalty also reduces the performance of SDM2 in predicting forward mutations and hence further work is required to improve the functional form of the disruption penalty term, possibly by accounting for the compensatory changes upon mutation occurring both in the interior and on surface regions of the proteins.

**Table 3.** Performance of SDM2 for the forward and reverse mutations and the comparison with other methods

	*R* ^a^	
Method	Forward	Reverse
SDM	0.50	0.19
PROTS	0.46	0.45
MUPro	0.97	0.01
I-mutant2.0	0.94	0.05

^a^Pearson product–moment correlation coefficient.

## SUMMARY

SDM2 has been tested on a wide range of datasets routinely used in the literature. The newly updated ESSTs, based on residue packing density, improved the overall performance of the method. Analysis of the use of residue packing density has shown an improved ability to classify disease and non-disease mutations (40) and hence SDM2 with the newly updated ESST based on packing density is likely to be a useful tool for understanding disease mutations (53–59) and to guide protein engineering. In addition to the prediction of single mutations, the webserver also provides the additional option to run predictions of a list of user submitted mutations. The updated method has been shown to perform better with the hypothetical reverse mutation in comparison to other well-known machine-learning methods. The SDM2 predictions reported are available for download from the webserver by following the link to the ‘Data sets’ page. The web interface and the usability of the server have been considerably improved and designed to be compatible with most commonly used modern web browsers. In the future, the SDM2 method will be expanded to predict the impact of mutations on protein–protein and protein–ligand interactions.

## Supplementary Material

Supplementary Data
