# Supplementary figures and images for: SDM: a server for predicting effects of mutations on protein stability
Source: Nucleic Acids Res. 2017 May 19;45(Web Server issue):W229–35. doi: 10.1093/nar/gkx439 (PMC5793720; doi:10.1093/nar/gkx439)

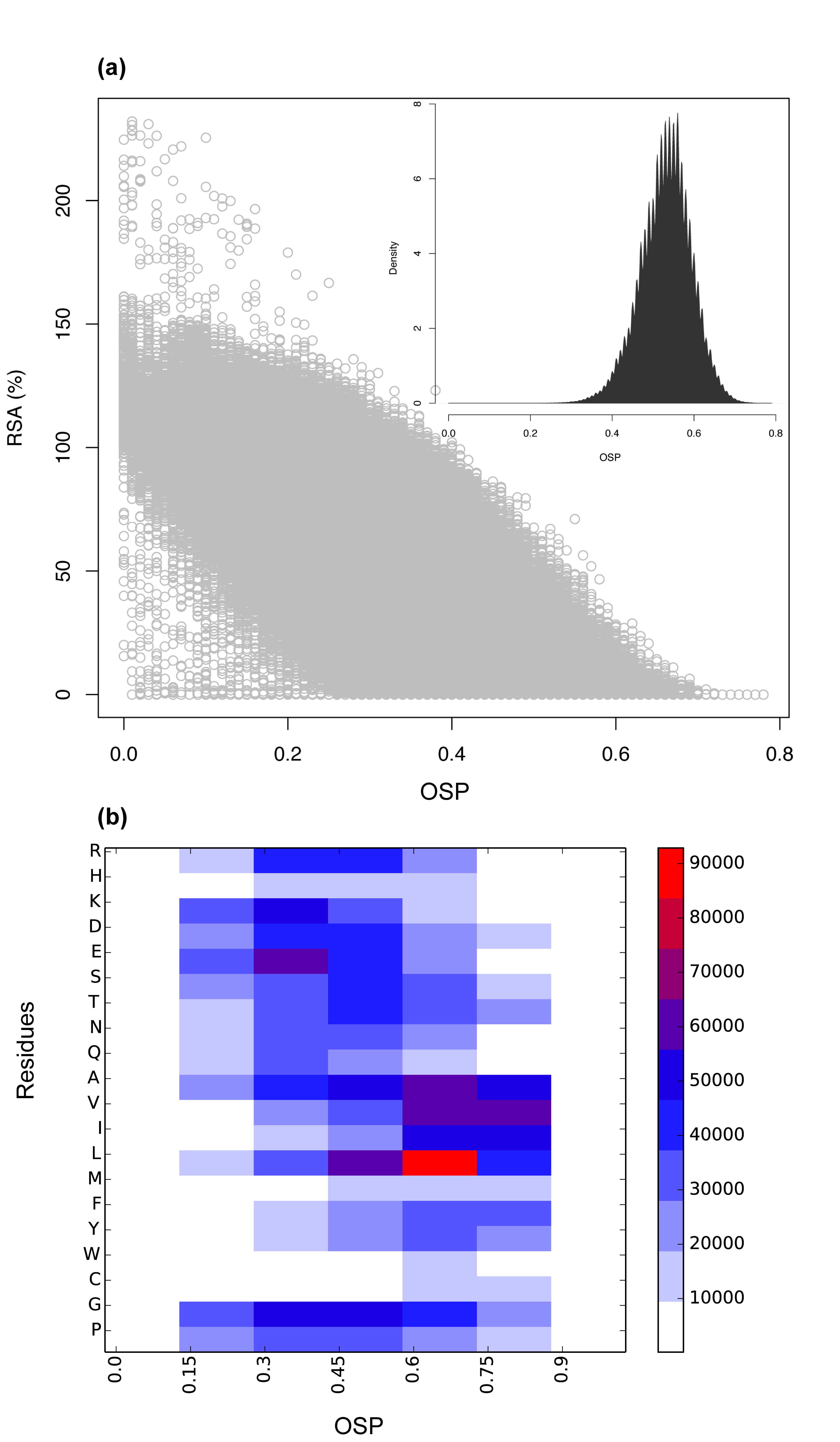

Supplement: Supplementary Data [file gkx439_supp.zip › nar-00456-web-b-2017-File009.tif]

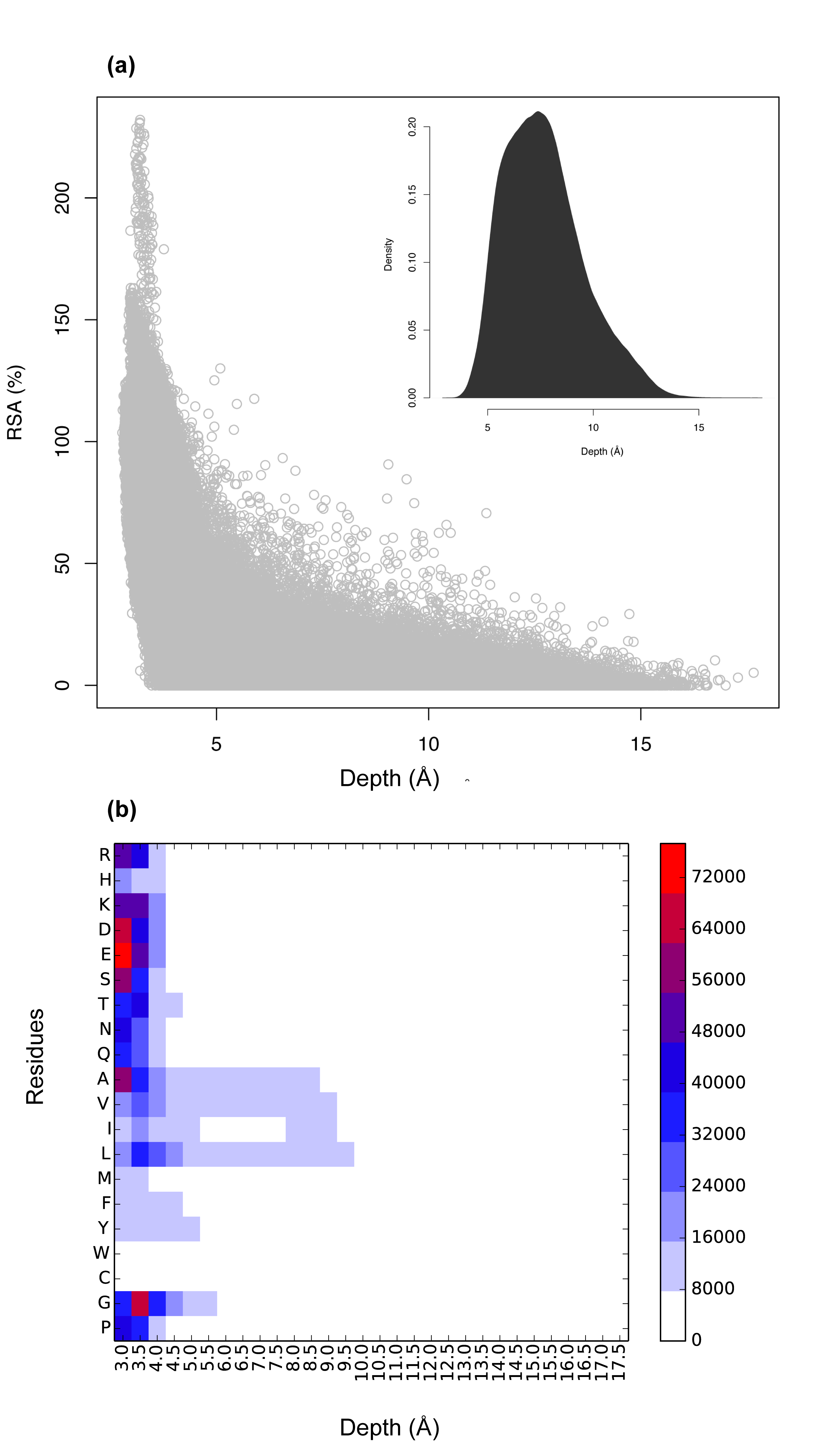

Supplement: Supplementary Data [file gkx439_supp.zip › nar-00456-web-b-2017-File010.tif]

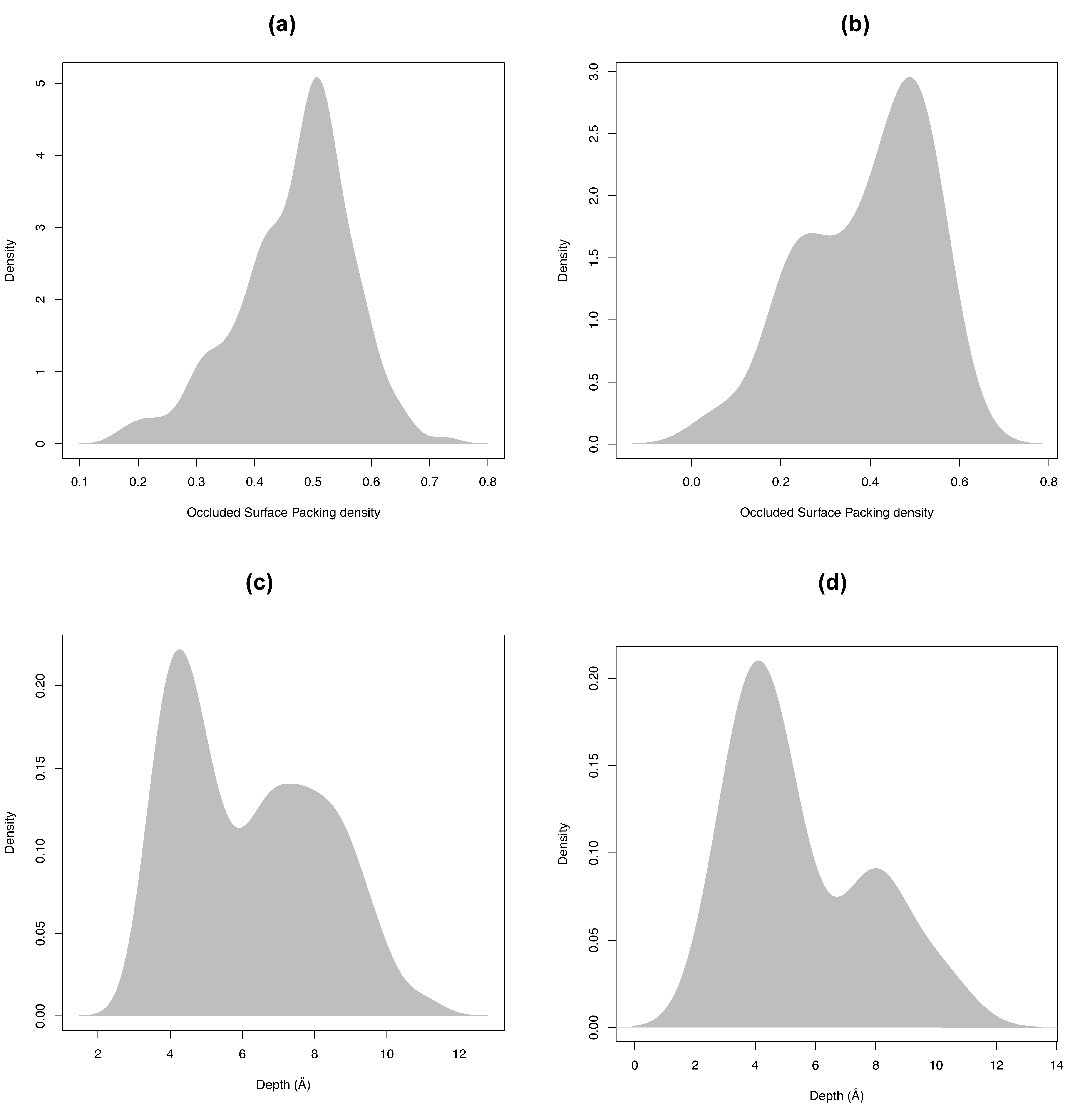

Supplement: Supplementary Data [file gkx439_supp.zip › nar-00456-web-b-2017-File011.tif]
