# Supplementary material for: SDM: a server for predicting effects of mutations on protein stability
Source: Nucleic Acids Res. 2017 May 19;45(Web Server issue):W229–35. doi: 10.1093/nar/gkx439 (PMC5793720; doi:10.1093/nar/gkx439)
Supplement: Supplementary Data [file gkx439_supp.zip › nar-00456-web-b-2017-File006.doc]

Table S1. Performance of SDM2 on the datasets using ESSTs based on different combinations of mainchain conformation angles, solvent accessibility, hydrogen bonding, occluded surface packing and residue depth.

| **ESST** | **R**^a^ | | |
| --- | --- | --- | --- |
|  | **P53** | **S350**^b^ | **S2648** |
| 216_RSA | 0.68 | 0.62/0.63/0.70 | 0.46 |
| 216_depth | 0.68 | 0.61/0.61/0.69 | 0.47 |
| 54_RSA | 0.62 | 0.60/0.60/0.67 | 0.46 |
| 648_RSA_OSP_depth | 0.66 | 0.62/0.62/0.69 | 0.48 |
|  | **Accuracy** | | |
| 216_RSA | 0.67 | 0.70/0.73/0.93 | 0.69 |
| 216_depth | 0.74 | 0.70/0.73/0.93 | 0.71 |
| 54_RSA | 0.67 | 0.70/0.73/0.92 | 0.69 |
| 648_RSA_OSP_depth | 0.74 | 0.70/0.73/0.92 | 0.71 |
|  | **MCC** | | |
| 216_RSA | 0.10 | 0.31/0.34/0.73 | 0.26 |
| 216_depth | 0.26 | 0.32/0.34/0.73 | 0.28 |
| 54_RSA | 0.14 | 0.32/0.34/0.67 | 0.24 |
| 648_RSA_OSP_depth | 0.26 | 0.31/0.34/0.69 | 0.28 |

^a^Pearson product-moment correlation coefficient.

^b^Three values are shown separated by slash. The first value corresponds to the whole validation set of 350 mutants. The second value corresponds to the 309 mutants with ΔΔG prediction available for all predictors. The third value corresponds to 87 mutants with the experimental ΔΔG values causes >2 kcal/mol change and for which a ΔΔG prediction is available from all predictors.

MCC is the Matthews Correlation Coefficient.

Please see Supplementary text for details about the calculation of Pearson product-moment correlation coefficient, Accuracy and MCC**.**
