# Supplementary material for: SDM: a server for predicting effects of mutations on protein stability
Source: Nucleic Acids Res. 2017 May 19;45(Web Server issue):W229–35. doi: 10.1093/nar/gkx439 (PMC5793720; doi:10.1093/nar/gkx439)
Supplement: Supplementary Data [file gkx439_supp.zip › nar-00456-web-b-2017-File007.doc]

Table S2. Correlation between SDM2 stability prediction and the experimental ΔΔG values for the datasets without the contribution from cavity penalty.

| **Dataset** | **R**^a^ |
| --- | --- |
| P53 | 0.61 |
| S350 | 0.55/0.55/0.62^b^ |
| S2648 | 0.46 |

**^a^**Pearson product-moment correlation coefficient.

^b^Three values are shown separated by a slash. The first value corresponds to the whole validation set of 350 mutants. The second value corresponds to the 309 mutants with ΔΔG prediction available for all predictors. The third value corresponds to 87 mutants with the experimental ΔΔG values causes >2 kcal/mol change and for which a ΔΔG prediction is available from all predictors.
