# Supplementary material for: SDM: a server for predicting effects of mutations on protein stability
Source: Nucleic Acids Res. 2017 May 19;45(Web Server issue):W229–35. doi: 10.1093/nar/gkx439 (PMC5793720; doi:10.1093/nar/gkx439)
Supplement: Supplementary Data [file gkx439_supp.zip › nar-00456-web-b-2017-File012.doc]

**SUPPLEMENTARY TEXT**

**METHODS**

**Definition of structural environment**

In this study the following structural parameters have been used to define the local structural environment of amino acid residues for the purpose of calculating environment specific substitution tables (ESSTs).

***(a) Mainchain conformation***

Based on the torsional angle, nine mainchain conformations were defined and classified in the following order as being α-helix, β-sheet, a, b, p, t, l, g or e. The program sstruc was used to calculate the mainchain conformational classes and is available as part of the JOY (a program to annotate protein sequence alignments with three-dimensional (3D) structural features) package (1)

***(b) Relative sidechain solvent accessibility (RSA)***

Three classes of RSA were defined based on the method of Lee and Richards (2). (a) RSA < 17% were defined as inaccessible, (b) RSA > 17% and RSA <=43% were defined as partially accessible and, (c) RSA > 43% were defined as accessible. Relative sidechain solvent accessibility was defined based on the method of Lee and Richards (2). The accessibility of each residue in a structure was calculated using the program psa which is available as part of the JOY package (1). The cut-off for RSA was selected based on the previous implementation of SDM webserver (3).

***(c) Residue occluded surface packing (OSP)***

A combination of relative sidechain solvent accessibility (RSA) and OSP (4,5) was used to define three classes residue packing. (a) Residues with RSA >= 7% were defined as solvent exposed, (b) Residues with RSA < 7% and OSP <= 0.56 were defined as moderately packed, and (c) Residues with RSA < 7% and OSP > 0.56 were defined as tightly packed. Relative sidechain solvent accessibility was defined based on the method of Lee and Richards (2). The cut-off for OSP is selected based on the observation that the core of the protein corresponds to the OSP value of 0.56 (5). The accessibility of each residue in a structure was calculated using the program psa which is available as part of the JOY package (1).

***(d) Residue depth***

A combination of relative sidechain solvent accessibility (RSA) and residue depth (6,7) was used to define three classes residue depth. (a) Residues with RSA >= 7% were defined as solvent exposed, (b) Residues with RSA < 7% and depth <= 6.0 Å were defined to be buried the residues at intermediate depth level, and (c) Residues with RSA < 7% and depth > 6.0 Å were defined to be the buried residues at higher depth level.

***(e) Hydrogen bonding***

Two different classes of hydrogen bonding have been used for the purpose of calculating ESSTs

(a) Eight class of hydrogen bonding were defined based on the sidechain hydrogen bonding with other sidechains, as well as the amides and carbonyls of the mainchain. This definition was used in the original implementation of SDM method (8). The hydrogen bonds were calculated using the program hbond between donor and acceptor atoms within 3.5 Å except for interactions involving sulphur atoms where 4.0 Å was used. The program hbond is available as part of the JOY package (1).

(b) Two classes of hydrogen bonding were defined based on the satisfaction of sidechain hydrogen bond. The satisfied and unsatisfied sidechain hydrogen bonds were determined based on the criteria described by Worth and Blundell (9).

**ESSTs**

Using the classification of the above structural parameters various combination of ESSTs could be computed. The following sets of ESSTs have been used in this study.

***(a) 216_RSA:*** Represents a total of 216 ESSTs obtained by the combination of mainchain conformation, RSA and sidechain hydrogen bonding (9 mainchain conformation class × 3 RSA class × 8 hydrogen bonding pattern).

***(b) 216_OSP:*** Represents a total of 216 ESSTs obtained by the combination of mainchain conformation, OSP and sidechain hydrogen bonding (9 mainchain conformation class × 3 OSP class × 8 hydrogen bonding pattern).

***(c) 216_depth:*** Represents a total of 216 ESSTs obtained by the combination of mainchain conformation, residue depth and sidechain hydrogen bonding (9 mainchain conformation class × 3 residue depth class × 8 hydrogen bonding pattern).

***(d) 54_RSA:*** Represents a total of 54 ESSTs obtained by the combination of mainchain conformation, RSA and sidechain hydrogen bonding satisfaction (9 mainchain conformation class × 3 RSA class × 2 sidechain hydrogen bonding pattern).

***(e) 648_RSA_OSP_depth:*** Represents a total of 648 ESSTs obtained by the combination of mainchain conformation, OSP, depth and sidechain hydrogen bonding (9 mainchain conformation class × 3 OSP class × 3 depth class × 8 hydrogen bonding pattern).

**Evaluation metrics**

In this section we describe the evaluation metric used in the study.

**Pearson’s correlation coefficient** **(R)**: The Pearson’s correlation coefficient is a measure of the linear dependence of two variables (in our case, experimental and predicted change in affinity or stability) and is defined as the covariance of the two variables divided by the product of their standard deviations.

**True positive (tp)** is an item correctly predicted as belonging to the positive class (e.g., a stabilizing mutation correctly assigned).

**False positive (fp)** is an item incorrectly predicted as belonging to the positive class (e.g., a stabilizing mutation incorrectly assigned).

**True negative (tn)** is an item correctly predicted as belonging to the negative class (e.g., a destabilizing mutation correctly assigned).

**False negative (fn)** is an item incorrectly predicted as belonging to the negative class (e.g., a destabilizing mutation incorrectly assigned).

**Accuracy:** is the fraction of correct results described as

$$Accuracy=\frac{tp+tn}{tp+tn+fp+fn}$$

**Matthews Correlation Coefficient (MCC):**

The Matthews correlation coefficient is a metric of the quality of a classifier that correlates the actual and predicted classes, taking into account all components of a confusion matrix. It is a balanced metric that can be used even for experiments with very different sized classes. MCC values range from -1 to 1, where 1 denotes a perfect classifier, 0 a random classifier and -1 a classifier in total disagreement with actual and predicted classes. From a confusion matrix, the MCC can be calculated as follows:

$$MCC=\frac{tp*tn-fp*fn}{\sqrt{(tp+fp)(tp+fn)(tn+fp)(tn+fn)}}$$

**RESULT**

**Analysis of the distribution of occluded surface packing, residue depth and relative sidechain solvent accessibility**

We analysed the distribution of RSA (8) with respect to OSP (4) and the depth of the residue (6) using the TOCCATA representative protein structures described above. The distribution of RSA and OSP reveals that the solvent inaccessible residue (RSA < 7%) exhibits varying levels packing (Figure S1a). The insert in Figure S1a shows frequency of OSP values for solvent inaccessible residues. The packing of buried residues was highly varied, ranging from tightly packed to surprisingly disperse residues with the peak around 0.53. This observation is in agreement with the OSP values (0.56) previously reported from the analysis of the core protein residues (5). The study has also reported an average OSP value of 0.33 for solvent exposed residues. The heat-map in Figure S1b reveals that the residue methionine is frequently found among other hydrophobic residues (Gly, Tyr, Phe, Ile, Val and Ala) in the tightly packed region of the protein (OSP > 0.56). Whereas the in the loosely packed and solvent exposed regions of the protein (OSP <= 0.33) the residues Gly, Ala, Lys, Glu, Asp and Arg were found to occur frequently with Glu being most frequent. Similar distribution analysis using RSA and depth (Figure S1a) shows that for solvent inaccessible residues the depth distribution is wider than its OSP counterpart with the minimum and maximum values of residue depth corresponding to 2.3 Å and 17.7 Å respectively. The average depth for inaccessible residues is around 8.0 Å. The heat-map in Figure S2b shows that the residues Gln, Asn, Thr, Ser, Glu, Asp, Lys and Arg are frequently found at depth level <= 3.5 Å. Residues Ala, Gly and Pro have also been frequently found at this level. Only residues Leu, Ile, Val and Ala are frequently found at higher depth >=8.0 Å.

**References**

1.	Mizuguchi, K., Deane, C.M., Blundell, T.L., Johnson, M.S. and Overington, J.P. (1998) JOY: protein sequence-structure representation and analysis. *Bioinformatics*, **14**, 617-623.

2.	Lee, B. and Richards, F.M. (1971) The interpretation of protein structures: estimation of static accessibility. *J Mol Biol*, **55**, 379-400.

3.	Worth, C.L., Preissner, R. and Blundell, T.L. (2011) SDM--a server for predicting effects of mutations on protein stability and malfunction. *Nucleic Acids Res*, **39**, W215-222.

4.	Pattabiraman, N., Ward, K.B. and Fleming, P.J. (1995) Occluded molecular surface: analysis of protein packing. *J Mol Recognit*, **8**, 334-344.

5.	Fleming, P.J. and Richards, F.M. (2000) Protein packing: dependence on protein size, secondary structure and amino acid composition. *J Mol Biol*, **299**, 487-498.

6.	Chakravarty, S. and Varadarajan, R. (1999) Residue depth: a novel parameter for the analysis of protein structure and stability. *Structure*, **7**, 723-732.

7.	Tan, K.P., Nguyen, T.B., Patel, S., Varadarajan, R. and Madhusudhan, M.S. (2013) Depth: a web server to compute depth, cavity sizes, detect potential small-molecule ligand-binding cavities and predict the pKa of ionizable residues in proteins. *Nucleic Acids Res*, **41**, W314-321.

8.	Topham, C.M., Srinivasan, N. and Blundell, T.L. (1997) Prediction of the stability of protein mutants based on structural environment-dependent amino acid substitution and propensity tables. *Protein Eng*, **10**, 7-21.

9.	Worth, C.L. and Blundell, T.L. (2009) Satisfaction of hydrogen-bonding potential influences the conservation of polar sidechains. *Proteins*, **75**, 413-429.

**TABLE AND FIGURE LEGENDS**

Table S1. Performance of SDM2 on the datasets using ESSTs based on different combinations of mainchain conformation angles, solvent accessibility, hydrogen bonding, and residue depth.

Table S2. Correlation between SDM2 stability prediction and the experimental ΔΔG values for the datasets without the contribution from cavity penalty.

Table S3. The impact of disruption penalty on the SDM2 predictions for the forward and reverse mutations.

Figure S1. (a) Scatter plot of the relative sidechain solvent accessibility (RSA) and the residue occluded surface packing density (OSP). The inset in (a) shows the distribution of OSP for solvent-inaccessible residues (RSA < 7%). (b) Heat map shows the frequencies of occurrences of amino-acid residues at various OSP bins.

Figure S2. (a) Scatter plot of the relative side chain solvent accessibility (RSA) and the residue depth. The inset in (a) shows the distribution of depth for solvent inaccessible residues (RSA < 7%). (b) A heat map showing the frequencies of occurrences of amino acid residues in various depth bins.

Figure S3. Distribution of occluded surface packing density (OSP) and residue depth for the wildtype found in the dataset S2648. (a) Distribution of occluded surface packing density (OSP) found in the highly destabilising mutants. (a) Distribution of occluded surface packing density (OSP) found in the highly stabilising mutants. (c) Distribution of residue depth found in the highly destabilising mutants and (d) Distribution of residue depth found in the highly stabilising mutants.
